# Comprehensive Spatial-Temporal and Risk Factor Insights for Optimizing Livestock Anthrax Vaccination Strategies in Karnataka, India

**DOI:** 10.3390/vaccines12091081

**Published:** 2024-09-22

**Authors:** Jayashree Anandakumar, Kuralayanapalya Puttahonnappa Suresh, Archana Veeranagouda Patil, Chethan A. Jagadeesh, Sushma Bylaiah, Sharanagouda S. Patil, Divakar Hemadri

**Affiliations:** 1 ICAR-National Institute of Veterinary Epidemiology & Disease Informatics, Bengaluru 560064, Karnataka, India; anandjayashree541@gmail.com (J.A.); archanapatil1107@gmail.com (A.V.P.); ajchethan9343@gmail.com (C.A.J.); ss.patil@icar.gov.in (S.S.P.); divakar.hemadri@icar.gov.in (D.H.); 2 M S Ramaiah Institute of Technology, Bengaluru 560054, Karnataka, India; sushma.b3@gmail.com

**Keywords:** anthrax, spatiotemporal pattern, machine learning, risk map, basic reproduction number, vaccination coverage, herd immunity, management

## Abstract

Anthrax, a zoonotic disease affecting both livestock and humans globally, is caused by *Bacillus anthracis.* The objectives of this study were the following: (1) to identify environmental risk factors for anthrax and use this information to develop an improved predictive risk map, and (2) to estimate spatial variation in basic reproduction number (Ro) and herd immunity threshold at the village level, which can be used to optimize vaccination policies within high-risk regions. Based on the anthrax incidences from 2000–2023 and vaccine administration figures between 2008 and 2022 in Karnataka, this study depicted spatiotemporal pattern analysis to derive a risk map employing machine learning algorithms and estimate Ro and herd immunity threshold for better vaccination coverage. Risk factors considered were key meteorological, remote sensing, soil, and geographical parameters. Spatial autocorrelation and SaTScan analysis revealed the presence of hotspots and clusters predominantly in the southern, central, and uppermost northern districts of Karnataka and temporal cluster distribution between June and September. Factors significantly associated with anthrax were air temperature, surface pressure, land surface temperature (LST), enhanced vegetation index (EVI), potential evapotranspiration (PET), soil temperature, soil moisture, pH, available potassium, sulphur, and boron, elevation, and proximity to waterbodies and waterways. Ensemble technique with random forest and classification tree models were used to improve the prediction accuracy of anthrax. High-risk areas are expected in villages in the southern, central, and extreme northern districts of Karnataka. The estimated Ro revealed 11 high-risk districts with Ro > 1.50 and respective herd immunity thresholds ranging from 11.24% to 55.47%, and the assessment of vaccination coverage at the 70%, 80%, and 90% vaccine efficacy levels, all serving for need-based strategic vaccine allocation. A comparison analysis of vaccinations administered and vaccination coverage estimated in this study is used to illustrate difference in the supply and vaccine force. The findings from the present study may support in planning preventive interventions, resource allocation, especially of vaccines, and other control strategies against anthrax across Karnataka, specifically focusing on predicted high-risk regions.

## 1. Introduction

Anthrax is a worldwide zoonotic disease caused by *Bacillus anthracis*, a rod-shaped, Gram-positive, and spore-forming soil-borne bacterium (*B. anthracis*) [1,2]. The pathogen is mainly transmitted to herbivorous mammals, and human beings are also at risk of becoming infected through contact with infected animals or contaminated animal products such as meat, hide, and wool [1,3,4]. Historically, anthrax has been a global menace to both developed and developing countries, causing sporadic epidemics of major public health concern apart from economic losses, notably in the livestock sectors [5]. Anthrax continues to affect many regions of the globe, including Asia [6,7,8], Australia [9], North and South America [10], and various European [11,12] and sub-Saharan African countries [13]. Anthrax is endemic in many parts of Asia, with significant documentation across the continent and higher disease incidence rates particularly in China and Mongolia as well as former Soviet Union nations, including Kazakhstan and Uzbekistan [14]. Among southern Asian countries, anthrax is more prevalent in India and Bangladesh [6,15,16].

Environmental factors such as temperature, rainfall, wind direction, moisture, and soil temperature, together with vegetation, aggravate the spatial distribution of anthrax [2,17]. The geographic distribution of anthrax is largely determined by the propensity for *B. anthracis* to form spores and survive in different environmental niches [5,18]. The suitable environmental factors and ingestion of spores by susceptible animals may lead to anthrax incidence [19,20]. Due to its high resistance against extreme temperature, radiation, and chemical substances, the spore could exist several decades in the environment [18,21,22,23]. The main way of transmission in animals is the intake of spores while grazing in areas where anthrax incidence has occurred before. The long-distance dispersal of spores is influenced by meteorological conditions, including floods and strong winds [24,25], as well as birds, scavengers, or biting flies act as biological carriers that play important roles in transmitting infectious agents [26,27,28,29]. Additionally, certain soil properties such as high moisture content and calcium availability in combination with organic matter and neutral to alkaline pH are characteristics correlated with the occurrence of anthrax [18,28]. Spores tend to accumulate in low-lying regions through runoff from rainfall and water streams, thereby impacting the spatial distribution of incidence [30].

While assessing areas with anthrax occurrence, some zones remain free of disease because of the environment being non-conducive to the growth of the pathogen or even due to herd immunity. However, the major concern is the under-reporting of cases, improper disposal of carcasses of infected animals, and poor health and infrastructure facilities, as well as improper vaccination coverage in outbreak areas [31,32,33]. In Karnataka, the anthrax vaccine administered is the ANTHRAX SPORE VACCINE, LIVE, IP. This vaccine contains live spores from the avirulent Sterne strain of *B. anthracis*, suspended in 50% glycerin saline. Each cattle dose includes 10 million spores of *B. anthracis*. It is used as prophylactic vaccination against anthrax in animals and is effective even during outbreaks; usually, ring vaccination is conducted for up to 5 years, covering a 5 km radius of outbreak areas. The vaccine is supplied in 100-dose bottles, with each 100 mL bottle intended for subcutaneous injection. Cattle, buffalo, and horses receive 1 mL, while sheep, goats, and pigs receive 0.5 mL, and injections are given by veterinary doctors with all precautionary measures. All healthy animals over six months old (cattle and buffalo) and over three months old (sheep, goats, and pigs) should be vaccinated annually before the monsoon season in endemic areas. The vaccine must be stored and transported at 2–8 °C and has a shelf life of 12 months under these conditions [34,35]. The disease regularly occurs in countries where animal vaccination is not widespread. Although vaccines and several effective antibiotics exist, treatment is usually scattered in the underdeveloped parts of the world where anthrax is common. Information on anthrax distribution is important in control and management strategies; for instance, targeted vaccination and the optimization of resource allocation can take place by prioritizing prevention and control strategies in high-risk regions. There are many studies on different spatial and temporal levels of most aspects of anthrax in India. These studies include research in Karnataka on the effects of the El Niño and La Niña phenomena on anthrax [36]; assessments of basic reproduction numbers to gauge the effects of precipitation on anthrax in Karnataka [37]; and analyses on the spatial and temporal patterns of anthrax in livestock [8,36], wildlife [38,39,40], and human populations [41].

Although such studies have advanced understanding of anthrax ecology, spread, and dynamics in both space and time, they still lack further insights into the possible effects of environmental factors on anthrax occurrence and resource allocation that would be needed for optimal vaccine distribution based on the disease burden for limiting the spread of anthrax. Hence, keeping all these limitations in view, the present study was carried out for the state of Karnataka using 11 important meteorological and remote sensing variables, 10 soil parameters, and 4 geographical parameters as predictor variables for the retrospective data from 2000 to 2023. We used several machine-learning models and adopted an ensembling approach for mapping the risk of anthrax among livestock based on key climatic variables. The present research had two-fold objectives: firstly, to assess the current distribution of anthrax incidence and project the future habitat suitability and occurrence of anthrax events based on environmental predictors; and, secondly, to compute the minimum vaccination coverage within the livestock population that could be applied to effectively prevent the spread of anthrax and eventually achieve a disease-free environment in the near future. A good understanding of the spatiotemporal patterns of anthrax risk and their determinants is very important in informing the surveillance, control, and prevention strategies that are some of the essential tasks by farmers and veterinary and public health personnel.

## 2. Materials and Methods

### 2.1. Study Area

Karnataka is the southwestern state of peninsular India, characterized by 11.5° N and 18.5° N latitudes and 74° E to 78.5° E longitudes. The state has 31 districts and 29,340 villages, with a varying landscape from the green Western Ghats to the coastal plains. There is a tropical to subtropical climate that supports rich biodiversity, particularly in the Western Ghats. In this regard, livestock farming is essential. Prominent ones include cattle, buffalo, sheep, goats, poultry, and pigs, with 29.00 million livestock animals. Karnataka is basically a leading milk-producing state, wherein dairy cooperatives play a major role in collection and distribution. Government initiatives were undertaken for improving livestock rearing, enhancing productivity, and lifting rural livelihoods.

### 2.2. Data Source

#### 2.2.1. Anthrax Incidence Data

Anthrax is a notifiable disease in India; all suspected and confirmed cases in animals and humans are to be reported. Hence, the system of surveillance for anthrax in animals and human populations is based on notification. District veterinary offices and animal health centers are required to report animal diseases to the State Department of Animal Husbandry and Veterinary Services, where these data are systematically maintained. Confirmation of anthrax cases usually includes clinical observation of symptoms, microscopic examination of blood smears, culture examination, and, more recently, PCR testing. Data from all the states are subsequently consolidated and sent to the National Animal Disease Referral Expert System v2 (https://nivedi.res.in/Nadres_v2/ohai/ accessed on 17 May 2024) developed by the ICAR-National Institute of Veterinary Epidemiology and Disease Informatics, Bengaluru, for electronic archival material. In this study, georeferenced anthrax data at the village level, incidence, and year and month of occurrence from 2000 to 2023 of livestock anthrax were extracted from the NADRES v2, and secondary data were sourced from the literature [42]. The anthrax incidence reported in various web sources, such as ProMED News, was gathered and cross-referenced with our dataset for accuracy and validity.

#### 2.2.2. Pseudo-Absence Data

A greater proportion of pseudo-absence to present data may influence model performance either in a positive or negative way. This will add biases to model inter-comparisons, for which prevalence should be maintained constant at an intermediate level. To avoid bias in the comparison, pseudo-absence data generation on livestock anthrax were made. In line with past research, a prevalence rate was set at 0.5 to ensure a balanced proportion of pseudo-absences in relation to presences in the dataset [43,44,45]. Following that, a buffer of 50–70 km around the occurrence points was created to prevent an overlap of any cells comprising both presence and absence data, as well as to make sure that those cells did not correspond to typical environmental conditions, as in previous methods [46]. Random selection of pseudo-absence points was performed in the whole background area; however, grid points lying in the buffer zone were excluded.

#### 2.2.3. Livestock Population Data

India has an abundance of livestock, with around 535.78 million animals overall, of which 192.49 million comprise cattle. There are 109.85 million buffalo, 9.06 million pigs, 148.88 million goats, 74.26 million sheep, 0.44 million Mithun and yaks, 0.34 million horses and ponies, 0.08 million mules, 0.12 million donkeys, and 0.25 million camels (Department of Animal Husbandry & Dairying (DAHD)—20th Livestock Census of India) in India. In Karnataka, the livestock population includes 8.47 million cattle, 2.98 million buffalo, 11.05 million sheep, and 6.16 million goats. The village-level livestock population data from Karnataka of four major species reported to be susceptible to anthrax, i.e., cattle, buffalo, sheep, and goats, were collected from the 20th livestock census of India for further analysis.

### 2.3. Risk Factors

#### 2.3.1. Meteorological Data

The meteorological variables utilized in this study were sourced from the Global Land Data Assimilation System (GLDAS version 2), available at https://ldas.gsfc.nasa.gov/gldas accessed on 20 May 2024 [47]. These variables include air temperature (°C), potential evaporation rate (W/m^2^), rainfall precipitation rate (kg/m^2^/s), specific humidity (kg/kg), surface pressure (Pa), and wind speed (m/s). The data available at a spatial resolution of 0.25° × 0.25° in the network common data format (netCDF) were extracted (Table 1). Subsequently, they were converted into CSV files using R Studio with packages ‘raster’, ‘rgdal’, ‘qdap’, ‘data.table’, and ‘ncdf4’ to facilitate further analysis.

#### 2.3.2. Remote Sensing Data

The satellite data utilized in this study were retrieved from the Moderate Resolution Imaging Spectroradiometer (MODIS) [48]. These include the enhanced vegetation index (EVI) and potential evapotranspiration (PET) with a 16-day interval at a resolution of 500 m, land surface temperature (LST) with an 8-day interval at a resolution of 1-km, normalized difference vegetation index (NDVI) with a 16-day interval at a resolution of 500 m, and potential leaf area index (LAI) with a 16-day interval at a resolution of 500 m. These parameters were obtained from image products such as MOD16A2, MOD11A2, MOD13A1, and MOD15A2H, which were available in Hierarchical Data Format (HDF) file format with various spatial and temporal resolutions (Table 1). To process these data, the R packages “gdalutils” and “modis” were utilized to extract information from HDF files and convert them into GeoTIFF files. Subsequently, the R package “raster” was employed to organize all variables into raster (grid) type files, with each predictor represented as a raster layer reflecting a specific variable of interest.

#### 2.3.3. Soil Profile Data

Variation in animal health across geographic zones is somehow linked to variation in soils and their properties [49]. Some animal infectious diseases can originate from particular soils, and more direct effects may be expected if the pathogen is able to survive, grow, and reproduce in the soil [50]. *B. anthracis* is an organism that can survive in soil for decades. The current study considered some of the available soil parameters to determine their effect on anthrax spore survival and germination. Soil temperature data (k) were retrieved from the National Oceanic and Atmospheric Administration (NOAA) at a spatial resolution of 1 km × 1 km in netCDF format. The soil moisture (kg/m^2^) was extracted from GLDAS at a 0.25° × 0.25° spatial resolution in the netCDF format. The database of Karnataka soil health data (ICRISAT Development Centre, Government of Karnataka, 2016) was used to obtain other soil parameters, including available potassium, phosphorus, boron, zinc, sulphur nutrient status, organic carbon, electrical conductivity, and soil pH. These data were downloaded in the available tab format and converted to the required format using the R program.

#### 2.3.4. Geographical Parameters

The current study included data on elevation extracted from DIVA-GIS (https://diva-gis.org/ accessed on 22 May 2024), roadways (National Highways), and waterbodies and waterways extracted from OpenStreetMap Data Extracts (https://download.geofabrik.de/asia/india.html accessed on 22 May 2024). The data available in a zone-wise manner for India were downloaded in shapefile (shp.) format. These shapefiles were merged using the geopandas and pandas packages in Python. These shapefiles were used for mapping anthrax incidence regions over all geographical parameters under study. QGIS was used to estimate the distance from actual disease incidence regions to locations of waterways, waterbodies, and roadways in an attempt to understand the influence of those parameters on anthrax incidence.

### 2.4. Data Pre-Processing and Feature Engineering

Data pertaining to incidence of anthrax cases and all other relevant risk variables from various electronic sources were procured. A monthly anthrax case incidence was estimated and utilized as the dependent variable. The risk parameters were used on a monthly basis as independent variables. Data pre-processing is an iterative process of significant importance that helps in the conversion of raw data into comprehensible and practical formats. Normally, a raw dataset exhibits incompleteness and inconsistencies, lacks patterns and trends, and contains a lot of errors. Data pre-processing was performed to deal with noise elimination, handle missing data, detect anomalies, and label encoding in the datasets before the commencement of machine learning modelling. Consequently, in this work, data pre-processing was treated in a structured manner and encompassed the four primary stages: data cleaning, integration, transformation, and reduction. The stages were brought forth in a coherent way to guarantee both the quality and relevance of data for subsequent analysis and modelling tasks. First of all, errors and inconsistencies in the dataset were identified and corrected through careful data-cleaning procedures dealing with duplicates, outliers, and missing values. Various techniques, such as removal, imputation, and transformation, were applied to ensure data integrity and consistency. During the process of data integration, information gathered from various sources was combined into one single dataset, thereby overcoming the difficulties in format, structure, and semantics. This facilitated the creation of a unified dataset for analysis and model development. Following integration, data transformation techniques were employed to prepare the dataset for analysis, including normalization and standardization, which standardized the data attributes and facilitated subsequent modelling tasks. Finally, data reduction techniques, including feature selection and extraction algorithms, were implemented to reduce the dimensionality of the dataset while retaining critical information. By adhering to this methodology, we ensured that the dataset was thoroughly processed, optimized, and primed for effective utilization in machine learning tasks.

### 2.5. Spatial and Temporal Distribution of Anthrax Incidence

The spatial incidence mapping in our study was carried out to visualize the distribution of anthrax disease across various districts and villages reported between 2000 and 2023. In the present study, the district-level cumulative anthrax incidences were calculated and plotted to illustrate the geographic spread and endemicity of anthrax incidence in the study area. This provided insight into the prevalence and distribution pattern of the disease. Additionally, we investigated the temporal distribution of anthrax incidence by estimating cumulative incidences on a yearly and monthly basis from 2000 to 2023. This analysis aimed to check any seasonal variation in the incidence of anthrax within the period under consideration. By plotting these cumulative incidences over space and time, we aimed to gain a deeper understanding of the spatiotemporal dynamics of anthrax incidence, which is very critical in informing timely interventions and public health strategies.

### 2.6. Spatial Autocorrelation-Hotspot Analysis

In its simplest expression, spatial autocorrelation is the relationship between variable values, mainly influenced by their proximity in two-dimensional space [51]. There are various approaches, like Moran -I statistics [52] or Getis–Ord Gi* index [36] for spatial autocorrelation analysis. This study used the local Getis–Ord Gi* index to identify local autocorrelation and find the differences of the neighboring cell values for a geographic area. This index was effective in detecting “hot spots” demonstrating positive autocorrelation and “cold spots” indicating negative autocorrelation [53]. The positive Z score indicates the presence of a hotspot; the negative Z score, a cold spot. A Z score near zero indicates no apparent spatial cluster. It is a standardized measure that explains the concentration or dispersion of the disease. The deviation from zero shows the intensity of clustering or dispersion. A positive value reflects clustering in areas of high prevalence, and a negative value represents dispersion in areas with low prevalence of the disease [54].

### 2.7. Space-Time Cluster Analysis

The spatiotemporal clustering of anthrax incidences was assessed using the scan statistic test in SaTScan software version 9.6 [55]. SaTScan uses moving windows of different diameters to identify spatial clusters in a study region. This tool also identifies temporal clusters and demarcates ellipses or circles whose size keeps changing dynamically in a three-dimensional study region. Clusters are reported for circles with observed values greater than the predicted values. For the SaTScan analysis, village-level longitude and latitude coordinates were obtained for conducting clustering of the dataset for each attribute that related disease activity, case versus control, including both temporal and spatial features. It was applied to a yearly case dataset, taking the total number of incidents for every epidemiological unit recorded for that particular year and adjusting it against its total population. For this reason, the significance level for appropriate cluster identification was set in advance at *p* ≤ 0.05.

### 2.8. Linear Discriminant Analysis

Linear discriminant analysis is a classification-based machine learning algorithm that is developed from the theory of Fisher’s linear discriminant. Risk parameters were analyzed in the process of discriminant analysis to establish a linear relationship among them, which in turn became a strong foundation towards the precise understanding of how the attribute impacts computation and assessment. In this study, SaTScan was applied for detection of the significant and non-significant space-time clusters to identify risk occurrences. Then LDA was used to examine the variation of the environmental risk factors in these identified regions. The binary clustering status variable was assigned by the clustering status, where status = 1 for clustered regions and status = 0 for non-clustered regions. In the present study, an LDA was carried out with a pre-determined level of statistical significance of *p* ≤ 0.05 for all variables.

### 2.9. Modelling Anthrax Niches through Machine Learning

Several machine learning algorithms were applied to accurately estimate the effect of significant environmental risk factors on disease prediction. A total of 11 machine learning models, including naive Bayes (NB), flexible discriminant analysis (FDA), random forest (RF), support vector machine (SVM), multiple adaptive regression splines (MARS), adaptive boosting (ADA), gradient boosting machine (GBM), artificial neural network (NNET), classification tree analysis (CT), generalized linear models (GLMs), and generalized additive models (GAMs), were trained and validated to determine disease risk.

### 2.10. Model Evaluation Criterion

In this research, predictions based on significant environmental predictor variables were generated using different model artifacts. Comprehensive sets of evaluation metrics, including the Cohen’s kappa (Heidke skill score), receiver operating characteristic (ROC) curve, true skill statistics (TSS), area under the ROC curve (AUC), accuracy, precision, sensitivity, specificity, F1 score, logistic loss (LOGLOSS), error rate, and Gini coefficient, were used to assess the discriminative capacity of the fitted models [56,57,58]. These metrics were utilized to evaluate the accuracy of the prediction models based on the presence (1) or absence (0) of data.

### 2.11. Ensemble Modelling Approach for Better Accuracy

This study aggregated the outcomes of separate forecasts from multiple model methods using a Raster Stack approach [59]. Instead of relying on one best model, it is recommended to make a prediction by combining results of multiple models that return scores between 0 and 1. Averaging these scores yielded the best prediction [56,60]. In the present study, the average model score was derived by considering models that met the following criteria for further assessment of disease risk: Kappa > 0.60, ROC > 0.90, TSS > 0.70, AUC > 0.90, accuracy > 0.80, precision > 0.90, sensitivity > 0.90, specificity > 0.70, F1 score > 0.90, LOGLOSS < 0.30, error rate < 0.20, and Gini coefficient > 0.80 [61,62,63]. This approach ensures a robust evaluation and aggregation of predictions for a more accurate risk assessment.

### 2.12. Basic Reproduction Number Estimation to Understand Anthrax Transmission Dynamics

The basic reproduction number (Ro) is a threshold index that describes the extent of pathogen transmission. It is defined as the average number of secondary cases generated by a single infected individual over its entire period of infectiousness when introduced into a completely susceptible population. The importance of Ro lies in its threshold value: if Ro > 1, then it indicates a high chance of the disease spreading, while values below 1 mean there is a low risk. Various approaches exist for estimating Ro, encompassing methods such as the attack rate (AR) [64], exponential growth rate (EG) [65], maximum likelihood estimation (ML) [66], and time-dependent method (TD) [67,68]. In this study, we employed the AR, EG, and ML methods to determine the basic reproduction number according to previous studies [36,69].

#### 2.12.1. Attack Rate Estimate (AR)

The proportion of a population eventually infected is called the attack rate (AR) [64,70]. The required assumptions are a homogeneous mixing, closed population, and no intervention during the outbreak. The advantage of this approach is its simplicity, helping to estimate the spread of disease and predict outcomes based on initial conditions. However, the model’s assumptions do not always hold true in reality. The basic reproduction number and AR are related through (Equation (1)):

(1) $$Ro=-\frac{log\left(\frac{1-AR}{So}\right)}{AR-\left(1-So\right)}$$

where So is the basic vulnerability rate of population.

#### 2.12.2. Exponential Growth Rate (EG)

The Exponential Growth (EG) method estimates the Ro by fitting an exponential growth curve to early outbreak data. It is simple and intuitive, as it directly links Ro to the rate of case increase, making it easy to understand. This method is particularly useful in the early stages of an outbreak, where exponential growth is often observed, and it is quick to compute [65,71]. However, it assumes exponential growth, which may not hold as the outbreak progresses or interventions are introduced. It is also sensitive to the quality of initial data and does not account for varying transmission rates across different populations. The EG method works best in the early phase of an epidemic, when data are available and growth is rapid. During the early stages of an epidemic, Ro was linked to exponential growth as (Equation (2)):

(2) $$Ro=\frac{1}{M\left(1-r\right)}$$

where r is the exponential growth rate and M is the moment-generating function of generation time distribution. To obtain the growth rate, r, Poisson regression was used [72].

#### 2.12.3. Maximum Likelihood (ML) Estimate

The maximum likelihood of White and Pagano is based on the assumption that the number of secondary cases generated by an affected individual is Poisson distributed, with R representing the expected value. Optimizing log-likelihood over an exponential growth phase yields R. Given observation of (N_0_,N_1_, …, N_T_) incident cases over consecutive time units and a generation time distribution w, R is estimated by maximizing the log-likelihood (Equation (3)) [66,67,70], as follows:

(3) $$LL\left(R\right)=\sum_{t=1}^{T}\log\left(\frac{e^{-\mu_{t}}\mu_{t}^{N_{t}}}{N_{t}!}\right)\;\;\mathrm{where},\;\mu_{t}=R\Sigma_{i=1}t_{N_{t-i}W_{i}}$$

Here again, the likelihood must be calculated on a period of exponential growth, and the deviance R-squared measure may be used to select the best period. No assumption is made on mixing in the population. This approach is statistically rigorous, offering reliable estimates when sufficient data are available, and it can accommodate various generation time distributions, making it flexible. It is particularly useful during the intermediate phase of an outbreak when exponential growth has slowed. However, it requires detailed and accurate data and can be computationally intensive due to the complex likelihood maximization process. It may also struggle with sparse data, leading to less reliable estimates. This method is best suited for mid-phase epidemics when more data are available and greater accuracy is needed.

The methodology applied can be seen in similar studies on *B. anthracis* [36,37,69], where similar techniques were followed for Ro estimation. After calculating Ro values using these methods, the highest value among all estimates is selected as the final Ro for a particular village. This maximum value is chosen because it represents the worst-case scenario for the basic reproduction number. Since Ro indicates the number of secondary cases generated by a single case, the highest value reflects the greatest transmission potential for the disease.

The computational models used for estimating the Ro offer more reliable results by accounting for various generation time distributions. They are also dynamic, allowing for the inclusion of intervention effects. However, these methods face limitations, such as unreliable estimates due to inaccurate or limited data during the early stages of an outbreak. Additionally, they require a clear understanding of the time-dependency of case numbers, which is not always available, and the outcomes are often highly sensitive to data noise. Calculations of the Ro were performed using the R statistical software, version 3.6.3.

### 2.13. Estimation of Herd Immunity Threshold (HIT)

Herd immunity is achieved when a significant portion of the livestock population, or the herd, is vaccinated, thereby offering protection to susceptible animals. The more animals that are vaccinated in the population, the lower the likelihood that an unvaccinated susceptible animal will encounter the infection. With a large number of immune animals, it becomes challenging for diseases to spread between individuals, effectively breaking the chain of infection. The herd immunity threshold refers to the proportion of the animal population that needs to be immunized against an infectious disease for the said disease to be stabilized within the herd or population. When this threshold is reached through vaccination, each case leads to precisely one more case, causing the infection to become stable in the population of livestock, i.e., Ro = 1. The HIT was determined according to previous methods [73,74] using the formula (Equation (4)):

(4) $$HIT=1-\frac{1}{Ro}$$

The value obtained can be used in the course of controlling anthrax disease through vaccination programs in livestock.

### 2.14. Determination of Vaccine Coverage (Vc)

Ro serves as a crucial factor in determining the minimum vaccination coverage needed for disease elimination within the livestock population in a specific geographical region. Utilizing the herd immunity threshold, which incorporates Ro values, the minimum vaccination coverage (Vc) necessary for controlling or eliminating livestock diseases was calculated for regions that are identified to be at anthrax risk, as previously documented [73,74] (Equation (5)). The following calculation provides essential insights into the vaccination strategies required to effectively combat diseases within the livestock population:

(5) $$Vc=\left(1-\frac{1}{Ro}\right)Ve$$

where Ve—vaccine efficacy level at 70%, 80%, and 90%

Vaccine efficacy typically depends on the type of vaccine and livestock population. Therefore, based on the calculated Ro values, the required vaccination coverage for anthrax disease was estimated by considering three scenarios of vaccine efficacy at 70%, 80%, and 90%.

### 2.15. Vaccination Supply and Demand Status in Karnataka

District-wise anthrax vaccination supply data from 2008–2022 were obtained from the State Department of Animal Husbandry and Veterinary Services, Bengaluru, Karnataka, India. The differences between the vaccination supply and actual vaccine requirements were compared to understand distribution of vaccine supply and strategizing optimal vaccine allocation based on the requirements.

### 2.16. Statistical Analysis and Packages

All the statistical analyses, risk mapping, and disease forecasting were conducted utilizing R statistical software version 3.1.3 (R Foundation for Statistical Computing, Vienna, Austria; version 3.4.3). R is a versatile platform for data mining, computation, and graphical presentation. It mainly relies on various R packages for data extraction, alignment, annotation, analysis, fitting, and model validation. As for the analysis, the present study used various R packages: plyr, dplyr, rgdal, raster, data.table, openxlsx, tmap, sp, spdep, sf, BAMM, foreign, geosphere, MASS, biomod2, dsimo, mgcv, randomforest, mda, gbm, and earth. In addition, hotspot and space-time cluster analyses were performed using Getis–Ord index and SaTScan v9, respectively.

## 3. Results

### 3.1. Spatial Distribution of Anthrax

**Figure 1:**
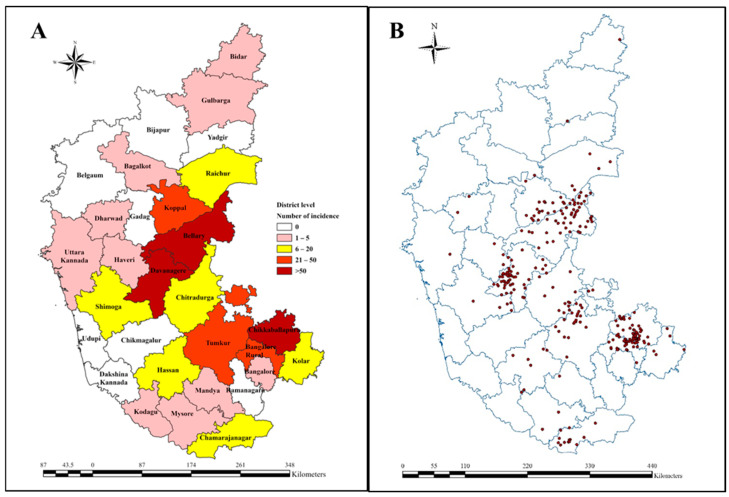
(**A**) Spatial map representing cumulative anthrax incidences recorded in Karnataka between 2000 and 2023, (**B**) Actual geolocations of villages where anthrax incidences were recorded between 2000 and 2023.

The district-wise cumulative anthrax incidence that occurred between 2000 and 2023 was determined and mapped to understand the spatial endemicity of anthrax in Karnataka (Figure 1A). The reported cumulative anthrax incidences were found to be the highest in the Bellary, Davanagere, and Chikkaballapura districts (range > 50 anthrax incidences), followed by the Koppal, Tumkur, and Bengaluru Rural districts (21–50 anthrax incidences), whereas in the Chamrajnagar, Chitradurga, Kolar, Hassan, Shimoga, and Raichur districts, 6–20 anthrax incidences were recorded. In districts such as Bengaluru Urban, Mandya, Mysuru, Kodagu, Uttara Kannada, Haveri, Dharwad, Bagalkot, Gulbarga, and Bidar, around 1–5 anthrax incidences were recorded. The actual geolocations of villages where anthrax incidences were recorded between 2000 and 2023 are presented in Figure 1B.

### 3.2. Temporal Distribution of Anthrax

**Figure 2:**
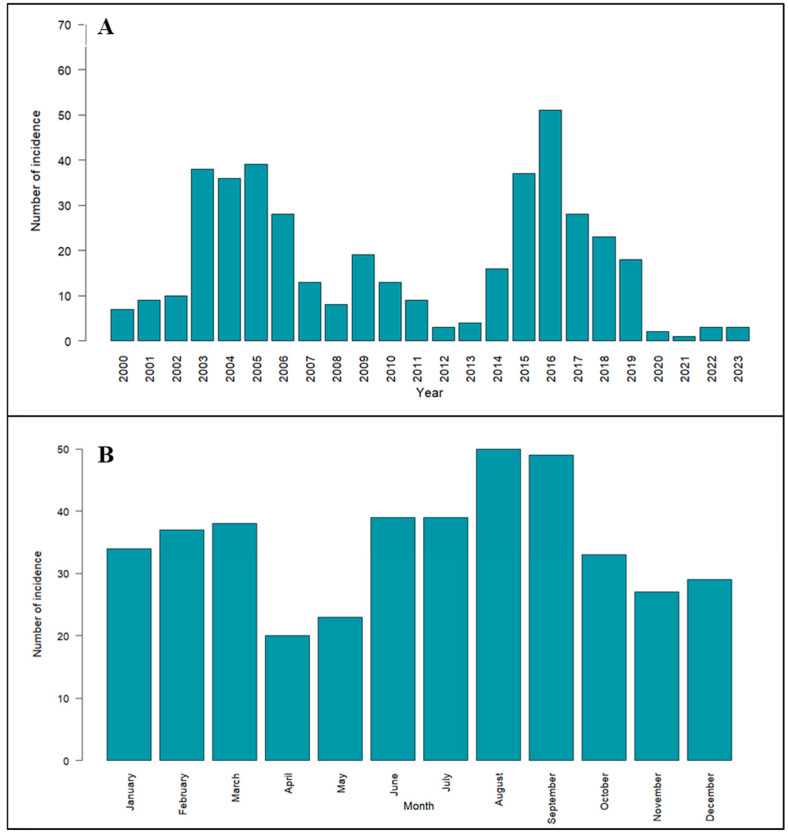
(**A**) Annual number of livestock anthrax incidences recorded in Karnataka during 2000–2023; (**B**) Cumulative monthly anthrax incidences recorded in Karnataka during 2000–2023.

The yearly distribution of anthrax incidence revealed significant peaks recorded between 2000 and 2023 (Figure 2A). A peak was observed between 2003 and 2005 with >30 recorded anthrax incidences, followed by a drastic decrease in anthrax incidences until 2014. One more peak was observed in 2015 with >30 anthrax incidences and >50 incidences in 2016, and a gradual decrease in anthrax incidences was noticed thereafter. An analysis of cumulative anthrax incidence reports from 2000 to 2023 revealed notable fluctuations across months (Figure 2B). The anthrax incidence in the study area was recorded throughout the year and was greater during the transition period from the dry to wet season, particularly from June to September, when precipitation and vegetation increase and the temperature starts declining after reaching maximum values (45 °C). Also, a greater number of anthrax incidences can be seen during January to March when most of the livestock animals migrate towards grazing areas located near waterbodies/waterways for feed and water purposes. These variations underscore the dynamic nature of anthrax incidence over time, highlighting the need for further investigation into contributing factors.

### 3.3. Spatial Autocorrelation Hotspot Analysis

**Figure 3:**
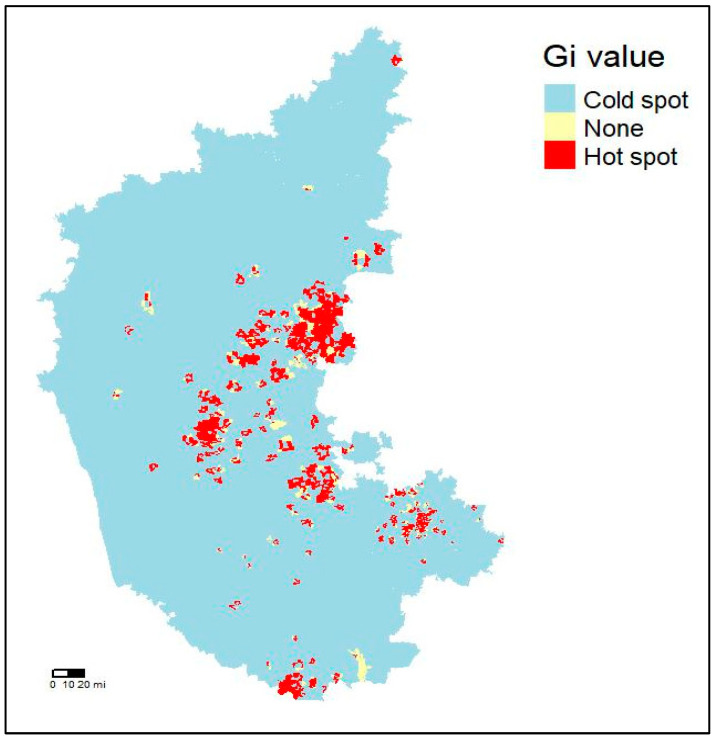
Hotspot map of anthrax in Karnataka identified through spatial autocorrelation.

The Getis–Ord Gi* index method serves to evaluate local spatial autocorrelation, thereby aiding in the identification of spatial clusters inherent within the dataset. The resultant output yields a Z score, wherein elevated values indicate the presence of hotspots or clusters. Positive and substantial Z scores denote statistically significant hotspots, whereas negative and lesser scores delineate cold spots. The Getis–Ord Gi* analysis is being utilized to identify villages with high risks of anthrax incidence for further analysis and modelling. A total of 1242 villages were identified as hotspots and are represented in Figure 3, and most of these villages were located in the central and southern districts of Karnataka.

### 3.4. Analysis of Space-Time Cluster 

Disease clusters were identified at the village level, with high disease incidences represented by red dots encircled by substantial red circles. In contrast, villages with low disease incidence disease clusters are depicted by blue dots within blue circles (Figure S1). A total of 15 significant clusters were identified within the time frame from 1 January 2000 to 31 December 2023; among these, 10 clusters (1–7, 9, 10, and 15) showed a high disease incidence comprising 275 villages and with a high relative risk (4.11–73.90) and LLR (9.94–57.64). A total of 5 clusters (8, 11–14) covering 13 locations were identified as low disease incidence clusters with relatively lower relative risk (0.06–0.15) and LLR (10.88–13.70) (Table 2; Figure S1).

### 3.5. Significant Risk Factors Identified through LDA

**Figure 4:**
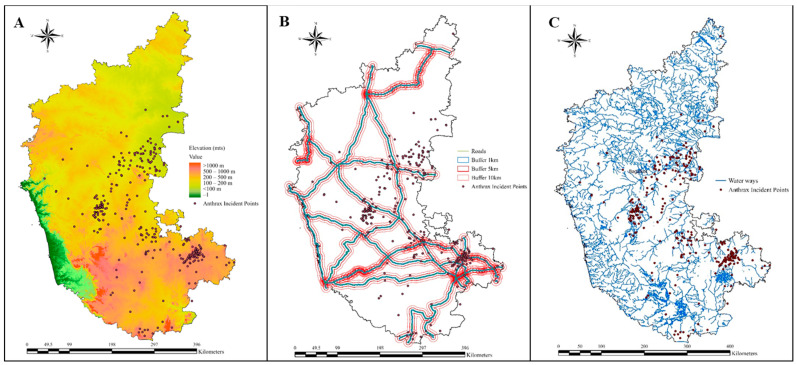
The map depicting anthrax incidence points plotted over (**A**) Elevation, (**B**) Roadways, and (**C**) Waterways.

The statistical analysis, particularly focusing on variables with a *p* value of 0.05 or lower, revealed significant correlations with disease occurrence, thus underscoring their potential impact on disease dynamics. Through rigorous linear discriminant analysis, several risk variables were identified as having substantial associations with anthrax disease incidence. Notably, climatic variables such as air temperature, surface pressure, LST, EVI, and PET; soil parameters such as soil temperature, soil moisture, pH, available potassium, sulphur, and boron; and geographical parameters such as elevation and distance from waterbodies and waterways to actual anthrax incidence points emerged as pivotal predictor variables of anthrax (Table 3). The anthrax incidence points plotted over elevation indicated that the incidence points were located at elevations > 100 m, and a majority of them were located at a higher elevation of >500 m (Figure 4A). The distance between anthrax incidence points and roadways at buffers of 1 km, 5 km, and 10 km revealed that the majority of points were within buffer zones of 5–10 km, and clusters of anthrax incidence points were observed in cross-sectional areas of national highways (Figure 4B). Figure 4C illustrates that the majority of the anthrax incidence points were located along the waterways. From this, we can conclude that elevation, roadways, and waterways coupled with significant environmental variables play a crucial role in anthrax occurrence and spread.

### 3.6. Anthrax Risk Assessment and Estimation through Machine Learning

**Figure 5:**
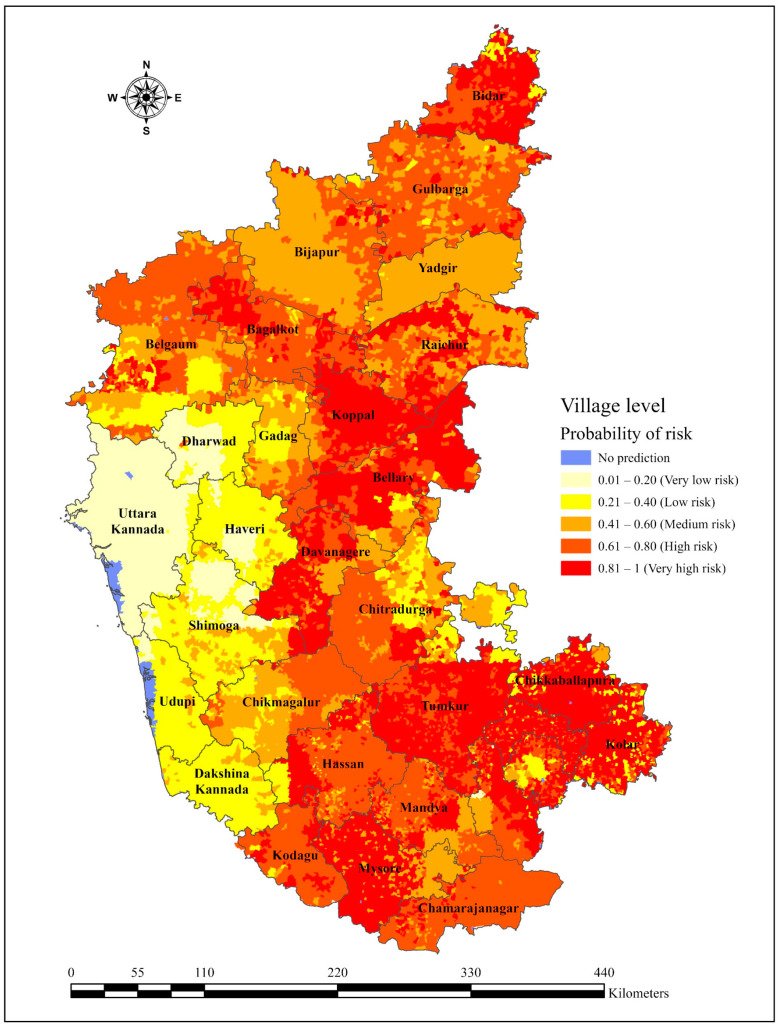
Predicted village-level risk map of anthrax in Karnataka showing variations in color, ranging from yellow (low risk) to red (high risk).

This research utilized climate-disease modelling to analyze significant environmental risk factors identified through LDA. Figure S2 visually displays the distribution pattern of anthrax-affected (case) and unaffected (control) regions, wherein red circles indicate areas with disease incidences and blue dots indicate regions without reported cases. Subsequently, case–control data, along with identified significant environmental variables, were fit through various machine learning models. The RF model proved superior performance with the Kappa (0.68), ROC (1.00), TSS (0.98), AUC (1.00), accuracy (0.99), precision (0.98), sensitivity (1.00), specificity (0.93), F1 score (0.99), LOGLOSS (0.12), error rate (0.01), and Gini coefficient (1.00), followed by CT model with Kappa (0.62), ROC (0.92), TSS (0.73), AUC (0.92), accuracy (0.86), precision (0.93), sensitivity (0.91), specificity (0.71), F1 score (0.92), LOGLOSS (0.27), error rate (0.14), and Gini coefficient (0.89). These two models emerged as highly accurate predictors that met stringent evaluation criteria and were considered for the ensemble approach (Table 4). To enhance confidence in the predictive outcomes, an innovative ensemble approach was employed by averaging the scores of the RF and CT models. This technique refined the prediction accuracy, enabling the delineation of anthrax risk within the study area. The combined prediction outcomes of several models were used in the current study, which are in the scale of 0 to 1 viz. (i) 0.01–0.20 = Very low risk, (ii) 0.21–0.40 = Low risk, (iii) 0.41–0.60 = Medium risk, (iv) 0.61–0.80 = High risk, and (v) 0.81–1.00 = Very high risk. The resulting risk map indicates that most of the villages in Bengaluru Rural, Chikkaballapura, Kolar, Ramnagara, Mandya, Mysuru, Chamrajnagara, Chikmagaluru, Kodagu, Hassan, and Davanagere in the southern and central regions and Koppal, Raichur, Bellary, and parts of Bagalkot, Gulbarga, and Bidar in the northern region of Karnataka are at medium to very high risk of anthrax occurrence (Figure 5).

### 3.7. Transmission Dynamics

Following the risk mapping, the basic reproduction number (Ro) was computed. Ro values exceeding 1.00 indicate regions or districts experiencing an upwards trend in disease incidence, while values below 1.00 suggest a decline in anthrax incidence in those regions. The village level Ro was estimated to range from 0.75 to 2.10, and a total of 245 villages were identified with Ro values > 1.00. The district-level mean Ro was worked out, and the lowest mean Ro was observed in Gulbarga (0.76) and the highest in Davanagere (2.25). A total of 20 districts were identified with mean Ro > 1.00, of which 11 districts were estimated to be at higher risk, with mean Ro > 1.50 (Table S1). The analysis highlights the areas at risk for anthrax transmission, offering valuable insights for implementing targeted surveillance and intervention strategies in these high-risk regions.

### 3.8. Herd Immunity Threshold

Herd immunity threshold (HIT) levels for anthrax in regions with an R₀ greater than 1.00 were estimated to range from 11.24% in Kolar to 55.47% in Davanagere. These HIT values represent the percentage of the animal population that must be immune to prevent the spread of anthrax among livestock. For instance, in Davanagere, an HIT of 55.47% means that 55.47% of the total livestock population needs to be immune to stop the transmission of anthrax (Table S1). However, controlling anthrax in developing countries like India faces significant challenges, including inadequate vaccination coverage, limited financial resources, and insufficient infrastructure, all of which hinder the development of herd immunity [75]. Achieving HIT levels through vaccination is crucial to controlling the spread of infection and preventing the incidence of livestock disease.

### 3.9. Vaccination Coverage

**Figure 6:**
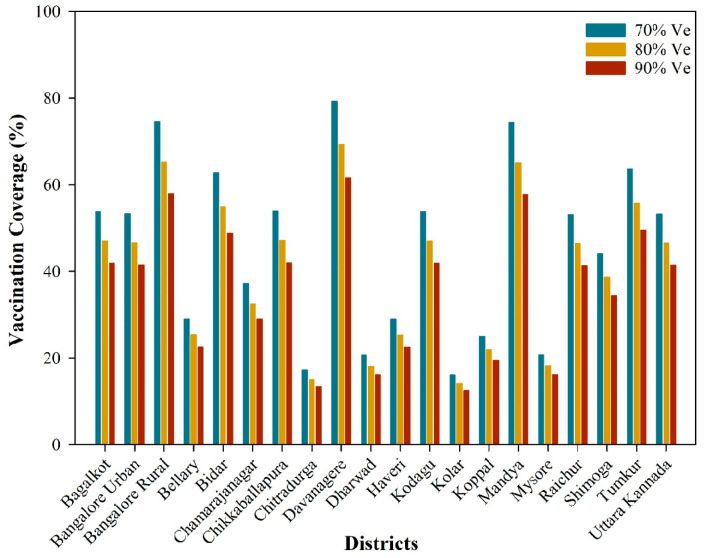
Assessment of vaccination coverage at different vaccine efficacy levels.

The vaccination coverage required for anthrax disease at different vaccine efficacy levels is depicted in Figure 6 and Table S1. This study considered three scenarios with vaccine efficacy levels of 70%, 80%, and 90%, as the exact efficacy or effectiveness of the anthrax livestock disease vaccine was not definitively known. The necessary vaccination coverage population in districts with Ro > 1.00 ranges from 16.06–79.24%, 14.05–69.34%, and 12.49–61.63% of the total livestock population at 70%, 80%, and 90% of vaccine efficacy levels, respectively (Table S1). The level of vaccination coverage needed is influenced by vaccine efficacy, which denotes the extent to which vaccinated animals become immune to anthrax disease, thereby limiting further spread or secondary infections. Vaccine efficacy is influenced by various factors, such as type of vaccine, timing of vaccination, duration of immunity, and vaccination program administered to the animals. Generally, greater vaccination coverage is required when vaccine efficacy is lower, as these two factors are inversely related (Figure 6).

### 3.10. The Vaccine Supply and Demand Trend in Karnataka 

**Figure 7:**
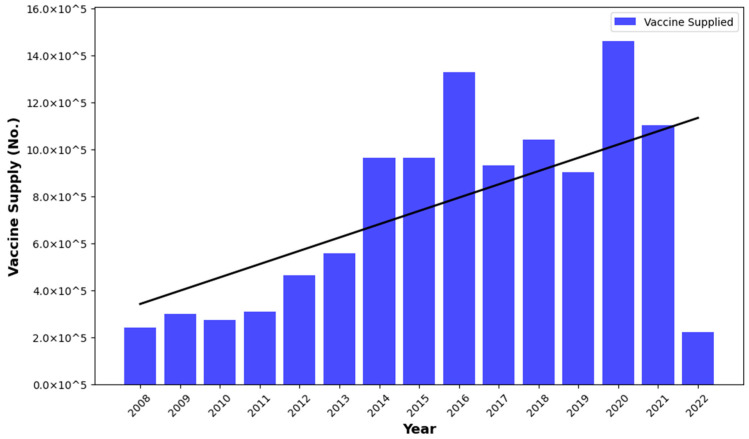
Vaccination supply trend in Karnataka (2008–2022).

Figure 7 depicts the yearly provision of anthrax vaccinations in Karnataka from 2008 to 2022, revealing a swift increase from 2014. The highest supply of anthrax vaccine was recorded in 2016 and 2020, indicating a notable escalation in immunization efforts. However, compared to previous years, 2022 saw a significant decline, suggesting shifts in vaccination strategies or challenges in the supply chain.

**Figure 8:**
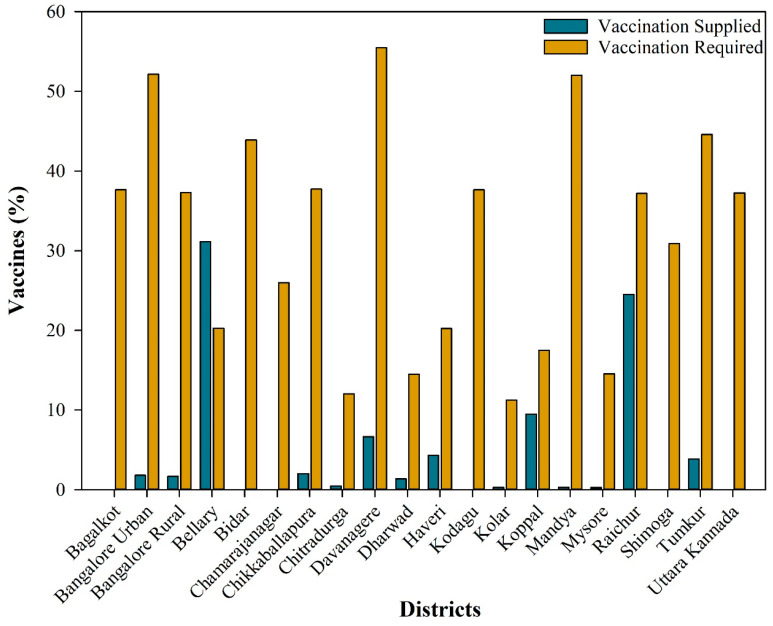
Differences in vaccines supplied vs. vaccines required to achieve herd immunity thresholds in high-risk anthrax districts in Karnataka.

In the ongoing vaccination campaign across various districts of Karnataka, substantial disparities in vaccine supply and demand have been noted. For instance, districts such as Bengaluru Urban, Bengaluru Rural, Chikkaballapura, Chitradurga Davanagere, Dharawad, Haveri, Kolar, Koppal, Mandya, Mysuru, Raichur, and Tumkur have received vaccines covering only a small percentage of the total livestock population, while the demand stands significantly higher as per herd immunity threshold level required, indicating an inadequate supply of vaccines to meet the local demand. Conversely, Bellary received relatively ample vaccine supplies compared to their requirements, suggesting potentially better vaccination coverage in this area. Some districts, such as Bagalkot, Bidar, Chamrajnagara, Kodagu, Shimoga, and Uttara Kannada, received no vaccines despite their requirements (Figure 8). However, this discrepancy in supply leads to an uneven distribution of vaccines and indicates inefficient resource allocation. Therefore, the estimated herd immunity threshold and vaccination coverage rates at different levels of vaccine efficacy in the present study will guide stakeholders or authorities in strategizing resource allocation for anthrax control.

## 4. Discussion

Anthrax is an endemic and widespread disease in Karnataka; therefore, there is a need to adopt practical strategies for identifying and prioritizing areas for control measures. The basic measures adopted by most countries for controlling anthrax have included regular vaccination, raising public awareness, and enforcing rigorous quarantine measures [76,77]. One of the vital methods for reducing the susceptibility of livestock to anthrax infection is vaccination. It is through anthrax vaccines that authorities intend to establish immunity in livestock populations against anthrax, thereby reducing the likelihood of transmission and outbreak of the disease. To achieve optimal vaccination coverage is often a logistically and operationally difficult task when dealing with large geographic areas and huge livestock populations.

In this respect, the present study underscores the role of environmental factors as a key tool for monitoring anthrax incidence. Traditionally, disease prediction research has predominantly relied on conventional statistical models, each exhibiting varying degrees of predictive accuracy [78,79,80]. However, our study highlights the pivotal role of climate and environmental factors in shaping the geographic dispersion of anthrax through the utilization of machine learning techniques. This, in turn, facilitates the identification of anthrax hotspot areas and informs decision-making to implement vaccination strategies and the estimation of herd immunity thresholds alongside the requisite vaccination coverage across varying levels of vaccine efficacy.

A systematic review on the spatiotemporal distribution and risk mapping of human anthrax has been conducted in which the authors reflected on conducting similar studies for livestock anthrax [41]. Some of the earlier studies have attempted to address this, but generally with limited datasets. Hence, in contrast to those, the present study depended on a large dataset from 2000 to 2023 for analyzing the spatiotemporal distribution, risk mapping, and vaccination coverage of livestock anthrax. This endeavor is useful in surveillance planning, and the findings warrant enhanced surveillance for livestock anthrax and focused vaccination in areas under high risk.

In this study, spatiotemporal analysis has shown that, from the year 2000 to 2023, there are dynamic patterns of anthrax incidence across different regions in the state of Karnataka. This allows for identification of geographical regions and time periods at increased risk by applying spatial analysis techniques such as hotspot identification and cluster analysis. While the prevalence of anthrax is there throughout Karnataka, it is still evident from our spatial distribution map, hotspot map, and space-time cluster analysis map that more endemicity is in the southern, central, and uppermost northern regions. This might be attributed to the warm, humid climate and alkaline soil conditions, which would favor spore survival and germination [2,77,81,82]. *B. anthracis*, being an extracellular pathogen, replicates rapidly in the blood and causes disease. Temporally, the year 2016 recorded remarkable anthrax incidences compared to the rest of the years, with June to September showing a high number of incidences, highlighting the need for increased vigilance and specific time-oriented interventions in order to curb the risks of anthrax outbreaks [5,77,81]. Furthermore, the integration of environmental risk factors, including climate and soil conditions, enhances understanding of anthrax transmission dynamics. Statistical analyses revealed significant associations between environmental variables and anthrax occurrence, with key predictor variables identified as air temperature, surface pressure, LST, EVI, and PET, soil parameters such as soil temperature, soil moisture, pH, available potassium, sulphur, and boron, and geophysical parameters such as elevation and distance from waterbodies and waterways to actual anthrax incidence points. These findings align with literature suggesting high anthrax incidence during dry and warm periods following intensive precipitation [5]. Our findings corroborate earlier reports indicating LST as a significant factor linked to anthrax incidence, with higher occurrences observed during the monsoon months of August, September, and October [78]. Additionally, a study highlighted air temperature and potential evaporation rate as potential risk indicators during El Niño years, while during La Niña years, air temperature, EVI, NDVI, specific humidity, and wind speed emerged as significant contributors to anthrax disease in Karnataka [36]. Soil pH, organic carbon, calcium, potassium, and zinc concentrations are believed to correlate with spore survival, while precipitation and wind speed aid in spore spread [2,81]. Animal contact with spores occurs through grazing grass close to the surface during low or scarce grass periods or by herds moving to restricted/endemic areas during water scarcity, increasing the likelihood of anthrax outbreaks [13,18]. These insights underscore the complex interplay between climatic variables and anthrax dynamics, emphasizing the need for nuanced surveillance and intervention strategies tailored to prevailing environmental conditions.

It has been evidenced in past years that machine learning models perform better than other statistical approaches for modelling areas [83,84]. In the present study, machine learning models RF and CT have shown robust predictive accuracy in unveiling intricate relationships between climatic variables and anthrax incidence. The ensemble approach, which averages scores from these models, further refines prediction accuracy and can be used to construct high-resolution risk maps. Our risk map, derived from the analysis, shows that anthrax is most likely to spread in the southern, central, and uppermost northern parts of Karnataka. These areas show heightened susceptibility to anthrax incidence, probably due to the suitable environment present in these regions, including warm and humid climates and alkaline soil, which would support the survival and germination of anthrax spores [2,13,36,81]. Other risk factors that might contribute to the raised risk noted in these regions include land use patterns, animal husbandry practices, and historical incidence of anthrax. These findings are consistent with several studies that have utilized similar machine learning approaches for predicting anthrax risk maps based on environmental variables in various regions, including Karnataka [36,37], India [81], Zimbabwe [13], North America [85], the Amhara regional state of Ethiopia [86], and the West African nation of Ghana [87].

The predicted risk maps, coupled with estimates of the basic reproduction number (Ro), provide actionable insight into targeted control measures for this disease. By identifying districts at elevated risk and estimating vaccination coverage necessary for effective disease control, authorities can prioritize resources and implement timely interventions in high-risk areas. Studies have documented that regular vaccination of ruminants in the endemic area of anthrax reduces the basic reproduction number of *B. anthracis* infection in Karnataka, India [36,37,88]. Inconsistency in vaccination administered and required can be the cause of a serious outbreak in areas that receive less vaccination than required. Hence, proactive vaccination campaigns among livestock in identified high-risk regions, as brought out by the present study, call for immediate attention towards policy and program. The anthrax incidence has been on a declining trend since 2016 (Figure 2A), which indicates that efforts undertaken by various vaccination programs in Karnataka are bearing fruits [88,89] and is consistent with the present study. The decline in anthrax vaccinations in 2022 may be primarily attributed to changes in vaccination. Karnataka follows a five-year ring vaccination program, covering a 5-km radius around endemic/outbreak villages [16]. As shown in Figure 2A, anthrax cases peaked in 2016, leading to intensive vaccination efforts until 2021 for a 5 year duration, as depicted in Figure 7. This reduced the need for large-scale vaccinations in 2022, which coincided with the overall decline in anthrax cases in the state in 2022 (Figure 2A). Furthermore, supply chain disruptions, particularly due to the COVID-19 pandemic, may have caused vaccine shortages or delays or misconceptions about vaccines, contributing to the lower vaccination numbers that year.

The efficacy of the vaccine typically varies depending on the type of vaccine, the strain used, and whether it is being administered to animals or humans [88]. It is important to note that if livestock are consuming feed containing antibiotics or are being treated with antibiotics individually, this can potentially render the vaccine ineffective [90]. The Food and Drug Administration (FDA) mandates controlled clinical trials and field studies to demonstrate vaccine efficacy. However, assessing the effectiveness of the anthrax vaccine presents challenges, as cases of inhalational anthrax are rare, even in regions where the disease is known to occur. Due to the infrequency of natural exposure, it is difficult to rely on real-world cases to evaluate the vaccine’s effectiveness [91]. The overall effectiveness of the anthrax vaccine in humans has been found to be approximately 92.5 percent [91]. However, due to variations in vaccine efficacy, the current study assessed vaccination coverage in animals at efficacy levels of 70%, 80%, and 90%. This vaccination coverage assessment helps to measure immunization levels, identify areas with low vaccination rates, and improve public health strategies.

There exist several strengths found in this study. First, we embedded a vast dataset spanning from 2000 to 2023, hence sweeping through all major trends in the incidence of anthrax within Karnataka. Furthermore, we validated the risk map using spatially independent data, which provided further strengthening of the analysis. Indeed, model evaluation is key, particularly for mapping to inform surveillance and activities related to the management of disease. Second, we estimated the requirements of herd immunity threshold and vaccination coverage under different levels of efficacy in high-risk regions of anthrax. Our study findings open up possibilities for reducing the spread and endemicity of anthrax in Karnataka. There are, however, some limitations to the study. Under-reporting and non-reporting of cases of anthrax during the period of study in Karnataka may modify the estimated basic reproduction number and in turn alter the herd immunity threshold and vaccination coverage required for a certain level of vaccine efficacy. Nevertheless, our estimate of vaccination coverage gives forewarning about the optimum population to be covered under vaccination, even in conditions of under-reporting or non-reporting. This study has therefore appreciably advanced the understanding of anthrax and identified the role of interdisciplinary approaches in understanding and managing this zoonotic disease. Spatial, temporal, and environmental data are combined with advanced analytical techniques in this contribution, putting forward an integrated framework for assessing anthrax risk and guiding evidence-based decision-making and vaccine coverage and resource allocation in disease control efforts.

## 5. Conclusions

This research underscores the crucial role of spatiotemporal analysis and environmental factors in monitoring anthrax outbreaks, emphasizing the need for targeted vaccination strategies in Karnataka. By integrating GIS with meteorological data, remote sensing, soil, and geophysical parameters, we have correlated these factors with anthrax occurrence to create detailed predictive risk maps. These maps help estimate key epidemiological metrics such as the basic reproduction number (Ro), herd immunity thresholds, and required vaccination coverage at various efficacy levels. The risk maps and projections serve as essential tools for optimizing resource allocation, particularly vaccine distribution. Additionally, they provide critical insights into potential future behavior, enabling informed planning and evaluation of strategies. Early findings from this study can be leveraged by policymakers, veterinarians, and farmers to implement necessary public health measures to control anthrax spread. The estimates of herd immunity and vaccination coverage for high-risk areas, as identified in this study, will be pivotal in guiding policy decisions. These findings support the strategic allocation of resources for comprehensive disease surveillance, ensuring a steady supply of anthrax vaccines and promoting regular training for veterinarians and farmers. Moreover, this study emphasizes the importance of developing a robust early warning system and rapid response mechanisms using AI and machine learning tools to address gaps in public health efforts. Ultimately, this approach aims to reduce the risk of anthrax outbreaks and strengthen preventive measures.

## Figures and Tables

**Table 1:** Risk factor data and their sources used in the current study.

Variable	Source	Units/Range	Attribute	Resolution
Meteorological parameters				
Air temperature	GLDAS version 2 https://ldas.gsfc.nasa.gov/gldas accessed on 20 May 2024	k	netCDF	0.25° × 0.25°
Potential evaporation rate		W/m^2^	netCDF	0.25° × 0.25°
Rainfall precipitation rate		kg/m^2^/s	netCDF	0.25° × 0.25°
Specific humidity		kg/kg	netCDF	0.25° × 0.25°
Surface pressure		pa	netCDF	0.25° × 0.25°
Wind speed		m/s	netCDF	0.25° × 0.25°
Remote sensing parameters				
LST	MODIS https://ladsweb.modaps.eosdis.nasa.gov accessed on 21 May 2024	°C	Raster	1 km × 1 km
NDVI		−1 to 1	Raster	500 m × 500 m
EVI		−1 to 1	Raster	500 m × 500 m
PET		mm	Raster	500 m × 500 m
LAI		m^2^/m^2^	Raster	500 m × 500 m
Soil parameters				
Soil temperature	NOAA https://www.psl.noaa.gov accessed on 20 May 2024	k	netCDF	1 km × 1 km
Soil moisture	GLDAS version 2 https://ldas.gsfc.nasa.gov/gldas accessed on 20 May 2024	kg/m^2^	netCDF	0.25° × 0.25°
Soil pH	Karnataka soil health data (ICRISAT Development Centre, Government of Karnataka) https://doi.org/10.21421/D2/QYCEGR accessed on 22 May 2024	Acidic < 6.5 Neutral 6.5–7.5 Alkaline > 7.5	tab	NA
Organic carbon		%	tab	NA
Electrical conductivity		ds/m	tab	NA
Available potassium		ppm	tab	NA
Available phosphorous		ppm	tab	NA
Available zinc		ppm	tab	NA
Available sulphur		ppm	tab	NA
Available boron		ppm	tab	NA
Geographical parameters				
Elevation	DIVA-GIS (https://diva-gis.org/ accessed on 22 May 2024)	m	Shape file	NA
Roadways	OpenStreetMap Data Extracts (https://download.geofabrik.de/asia/india.html accessed on 22 May 2024)	m	Shape file	NA
Waterbodies		m	Shape file	NA
Waterways		m	Shape file	NA

netCDF: network common data format, NA: not available.

**Table 2:** Spatiotemporal clusters identified for anthrax through SaTScan.

Cluster	Latitude	Longitude	Radius (km)	Time Frame		O	E	O/E	RR	LLR	*p* Value
				From	To						
1	13.42	77.53	31.89	1 January 2000	31 December 2016	81.00	19.87	4.08	4.82	57.64	0.001
2	13.27	77.82	34.67	1 January 2003	31 December 2019	76.00	18.69	4.07	4.75	53.62	0.001
3	13.59	77.84	33.99	1 January 2003	31 December 2016	68.00	18.85	3.61	4.11	41.24	0.001
4	12.24	76.57	159.94	1 January 2001	31 December 2019	58.00	15.02	3.86	4.32	37.75	0.001
5	14.04	76.02	45.55	1 January 2001	31 December 2017	38.00	8.05	4.72	5.09	30.15	0.001
6	13.98	75.22	67.42	1 January 2000	31 December 2018	32.00	7.18	4.46	4.74	23.76	0.001
7	14.82	76.25	52.98	1 January 2017	31 December 2017	12.00	1.28	9.39	9.64	16.29	0.001
8	15.86	76.05	0.00	1 January 2020	31 December 2021	1.00	17.22	0.06	0.06	13.70	0.004
9	14.53	75.85	11.20	1 January 2017	31 December 2017	4.00	0.06	63.23	63.83	12.67	0.005
10	14.67	75.77	7.51	1 January 2017	31 December 2017	5.00	0.18	28.03	28.36	11.87	0.009
11	13.92	76.92	7.47	1 January 2016	31 December 2018	2.00	17.72	0.11	0.11	11.66	0.01
12	14.13	76.83	20.50	1 January 2008	31 December 2018	1.00	14.98	0.07	0.07	11.51	0.01
13	15.42	76.81	5.78	1 January 2014	31 December 2015	3.00	19.68	0.15	0.15	11.38	0.011
14	13.96	76.75	11.44	1 January 2018	31 December 2018	1.00	14.33	0.07	0.07	10.88	0.019
15	15.21	76.76	0.00	1 January 2003	31 December 2015	3.00	0.04	73.38	73.90	9.94	0.046

O: observed cases, E: expected cases, O/E: observed/expected incidences, RR: relative risk, and LLR: log-likelihood ratio.

**Table 3:** Significant predictor variables identified through linear discriminant analysis.

Variables	Mean	Min	Max	SD	F-Value	95% CI	*p*-Value
Meteorological parameters							
Air temperature	24.16	18.70	35.06	3.06	5.11	24.01–24.31	0.024
Potential evaporation rate	237.27	81.06	503.32	81.62	0.81	233.28–241.26	0.37
Rainfall precipitation rate	0.0000341	8.06 × 10^−9^	2.10 × 10^−4^	3.20 × 10^−5^	1.41	3.25 × 10^−5^–3.56 × 10^−5^	0.24
Specific humidity	0.01	0.00	0.02	0.00	3.03	0.01–0.01	0.08
Surface pressure	93,422.48	89,580.16	100,800.28	1863.83	30.70	93,331.31–93,513.64	5.54 × 10^−8^
Wind speed	3.49	1.31	8.25	1.28	0.18	3.42–3.55	0.67
Remote sensing parameters							
LST	33.34	22.13	49.85	4.86	11.50	33.10–33.59	0.001
NDVI	0.42	0.04	0.86	0.15	2.47	0.41–0.43	0.12
EVI	0.28	0.07	0.64	0.10	5.70	0.27–0.28	0.017
PET	1236.65	20.30	3276.50	1197.63	13.55	1178.07–1295.22	2.65 × 10^−4^
LAI	0.08	0.01	2.50	0.12	0.01	0.07–0.08	0.93
Soil parameters							
Soil temperature	298.20	293.00	307.39	2.81	15.72	298.06–298.33	8.72 × 10^−5^
Soil moisture	24.99	8.78	39.94	6.82	12.84	24.65–25.32	3.82 × 10^−4^
Soil pH	7.40	4.90	9.90	0.92	31.53	7.35–7.44	3.73 × 10^−8^
Organic carbon	0.52	0.10	1.32	0.24	1.58	0.51–0.53	0.21
Electrical conductivity	0.29	0.07	14.70	1.06	0.02	0.24–0.34	0.88
Available potassium	133.52	18.00	653.00	90.75	106.32	129.09–137.96	3.23 × 10^−22^
Available phosphorous	9.61	0.20	92.00	13.39	3.00	8.96–10.27	0.08
Available zinc	15.10	1.20	256.60	24.63	26.94	13.89–16.30	0.70
Available sulphur	0.68	0.20	4.80	0.44	0.15	0.66–0.70	3.38 × 10^−7^
Available boron	0.73	0.06	2.40	0.45	12.26	0.71–0.76	0.001
Geographical parameters							
Elevation	614.80	−1.00	1810.00	5304.33	23.61	5296.38–5312.28	1.71 × 10^−6^
Road ways	13,927.74	9.00	113,867.00	16,235.13	0.15	13,133.66–14,721.83	0.70
Waterbodies	2079.39	0.00	10,171.00	2119.20	3.77	1975.74–2183.05	0.05
Waterways	3379.35	14.00	13,873.00	2892.72	18.35	3237.87–3520.84	2.32 × 10^−5^

Min: minimum, Max: maximum, SD: standard deviation, CI: confidence intervals.

**Table 4:** Evaluation matrices of machine learning models employed in this study for anthrax risk prediction.

Models	Kappa	ROC	TSS	AUC	Accuracy	Precision	Sensitivity	Specificity	F1 Score	LOG LOSS	Error Rate	Gini Coefficient
GLM	0.35	0.84	0.51	0.84	0.76	0.85	0.96	0.41	0.91	0.36	0.24	0.69
GAM	0.35	0.84	0.51	0.84	0.76	0.85	0.96	0.41	0.91	0.36	0.24	0.69
RF	0.68	1.00	0.98	1.00	0.99	0.98	1.00	0.93	0.99	0.12	0.01	1.00
GBM	0.46	0.95	0.79	0.95	0.90	0.92	0.99	0.68	0.95	0.25	0.10	0.90
NNET	0.00	0.50	0.00	0.50	0.22	0.50	0.00	0.00	1.00	5.07	0.78	1.00
MARS	0.43	0.92	0.67	0.92	0.84	0.89	0.97	0.59	0.93	0.29	0.16	0.83
FDA	−0.01	0.50	0.00	0.50	0.78	0.78	1.00	0.00	0.88	7.64	0.22	0.99
CT	0.62	0.92	0.73	0.92	0.86	0.93	0.91	0.71	0.92	0.27	0.14	0.89
SVM	0.54	0.89	0.72	0.89	0.88	0.86	1.00	0.41	0.92	0.77	0.12	0.78
NB	−0.35	0.85	−0.09	0.85	0.20	0.11	0.01	0.79	0.01	7.03	0.80	−0.70
ADA	0.79	0.87	0.75	0.87	0.93	0.94	0.98	0.76	0.96	2.28	0.07	0.99

The GLM, generalized linear model; GAM, generalized additive model; RF, random forest; GBM, gradient boosting machine; NNET, artificial neural network; MARS, multiple adaptive regression splines; FDA, flexible discriminant analysis; CT, classification tree analysis; SVM, support vector machine; NB, naive Bayes; ADA, adaptive boosting; ROC, receiving operating characteristic curve; TSS, true skill statistics; AUC, area under the ROC curve; LOGLOSS, logistic loss.

## Data Availability

The data that support the findings of this study are available on https://www.nivedi.res.in/Nadres_v2/ohai/ accessed on 17 May 2024 (Anthrax incidence and vaccine data for Karntaka—password enabled and will be shared upon reasonable request), and the environmental factors used in the study are available on the public repository GLDAS version 2—https://ldas.gsfc.nasa.gov/gldas accessed on 20 May 2024, MODIS—https://ladsweb.modaps.eosdis.nasa.gov accessed on 21 May 2024, NOAA—https://www.psl.noaa.gov accessed on 20 May 2024, Karnataka soil health data (ICRISAT Development Centre, Government of Karnataka) https://doi.org/10.21421/D2/QYCEGR accessed on 22 May 2024, DIVA-GIS—(https://diva-gis.org/ accessed on May 2024), Open Street Map Data Extracts—https://download.geofabrik.de/asia/india.html accessed on May 2024.

## Supplementary Materials

The following supporting information can be downloaded at: https://www.mdpi.com/article/10.3390/vaccines12091081/s1, Table S1: District-wise estimated basic reproduction number, herd immunity threshold and vaccination coverage requirement at different vaccine efficacy levels. Figure S1: Space time clusters of anthrax in Karnataka. Figure S2: Anthrax attacks case-control data are depicted on a map of Karnataka. (A) Case data: red-colored circles denote locations where anthrax has been reported, (B) Control data: blue-colored dots denote locations where anthrax has not been reported, and (C) Case-control data: displays both the existence and absence of anthrax incidence.
