# Supplementary material for: Comprehensive Spatial-Temporal and Risk Factor Insights for Optimizing Livestock Anthrax Vaccination Strategies in Karnataka, India
Source: Vaccines (Basel). 2024 Sep 22;12(9):1081. doi: 10.3390/vaccines12091081 (PMC11435676; doi:10.3390/vaccines12091081)
Supplement: Supplementary file 1 [file vaccines-12-01081-s001.zip › vaccines-3179135-supplementary.pdf]

Table S1: District-wise estimated basic reproduction number, herd immunity threshold and vaccination coverage requirement at different vaccine efficacy levels.

District	Ro	HIT (%)	Vc (%)		
			70 % Ve	80 % Ve	90 % Ve
Bagalkot	1.60	37.65	53.79	47.06	41.83
Bangalore Urban	1.60	37.32	53.31	46.65	41.46
Bangalore Rural	2.09	52.17	74.52	65.21	57.96
Bellary	1.25	20.26	28.94	25.33	22.51
Bidar	1.78	43.89	62.69	54.86	48.76
Chamarajanagar	1.35	26.00	37.14	32.50	28.89
Chikkaballapura	1.61	37.75	53.93	47.19	41.94
Chitradurga	1.14	12.05	17.21	15.06	13.39
Davanagere	2.25	55.47	79.24	69.34	61.63
Dharwad	1.17	14.51	20.73	18.14	16.12
Haveri	1.25	20.22	28.88	25.27	22.46
Kodagu	1.60	37.65	53.79	47.06	41.83
Kolar	1.13	11.24	16.06	14.05	12.49
Koppal	1.21	17.50	24.99	21.87	19.44
Mandya	2.08	52.04	74.34	65.05	57.82
Mysore	1.17	14.55	20.79	18.19	16.17
Raichur	1.59	37.20	53.14	46.50	41.33
Shimoga	1.45	30.90	44.14	38.62	34.33
Tumkur	1.81	44.61	63.72	55.76	49.56

Where, Ro- Basic Reproduction Number, HIT - Herd immunity threshold, Vc-Vaccination coverage, Ve-Vaccine efficacy

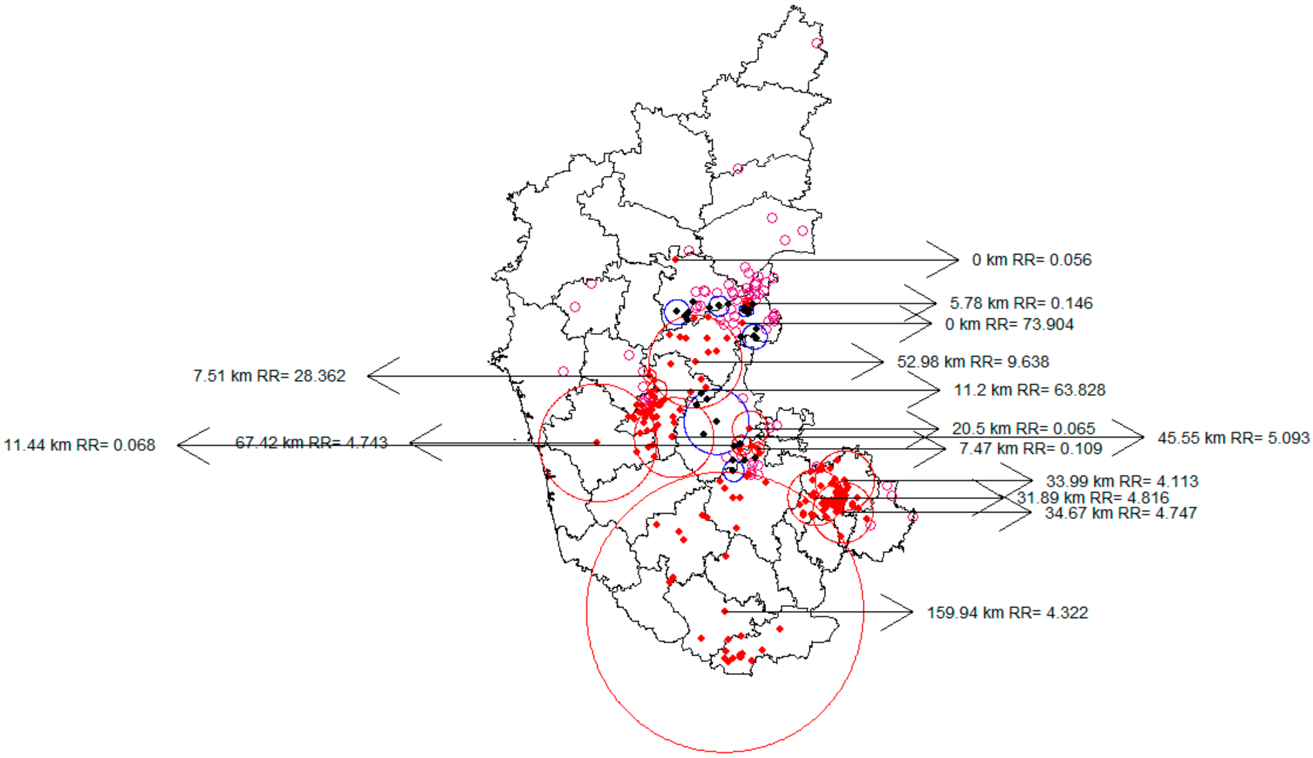

Figure S1: Space time clusters of anthrax in Karnataka

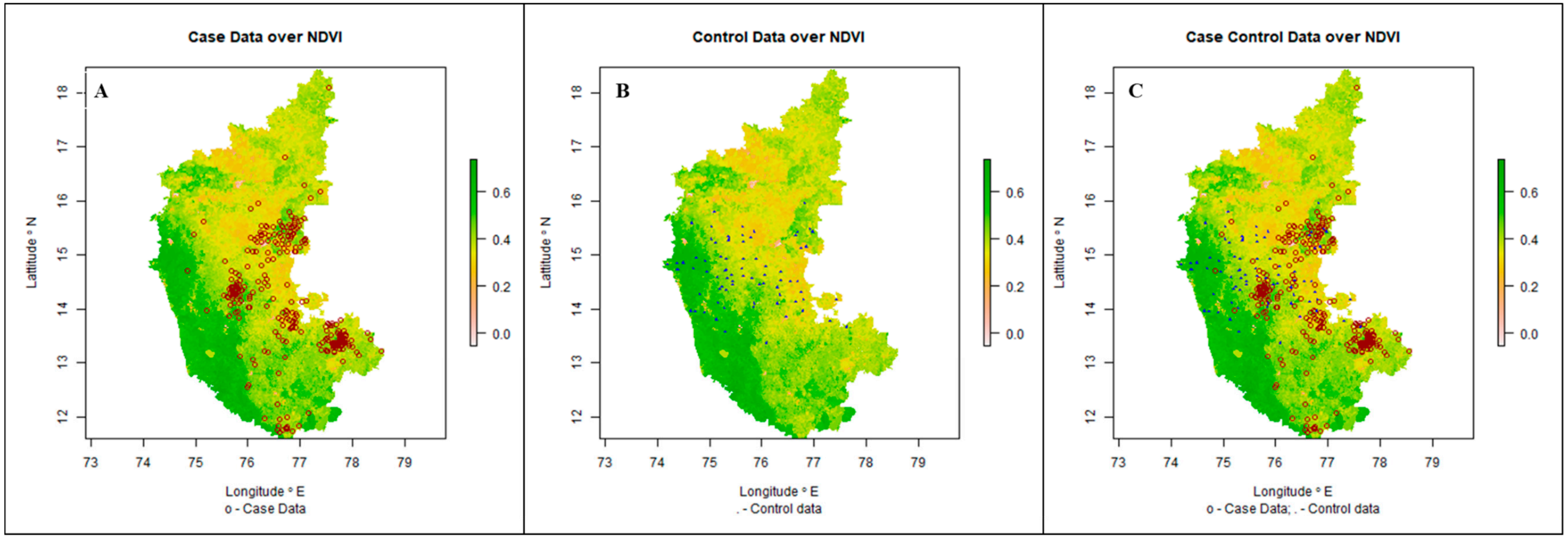

Figure S2: Anthrax attacks case-control data are depicted on a map of Karnataka (A) Case data: red-coloured circles denote locations where anthrax has been reported, (B) Control data: blue-coloured dots denote locations where anthrax has not been reported, and (C) Case-control data: displays both the existence and absence of anthrax incidence.
